# National trends in the prevalence of chronic kidney disease among Korean adults, 2007–2020

**DOI:** 10.1038/s41598-023-33122-1

**Published:** 2023-04-10

**Authors:** Soo-Young Yoon, Hye Won Park, Hyeon Jin Kim, Andreas Kronbichler, Ai Koyanagi, Lee Smith, Jae Il Shin, Sang Youl Rhee, Seung Won Lee, Jin Sug Kim, Hyeon Seok Hwang, Dong Keon Yon, Kyunghwan Jeong

**Affiliations:** 1 Division of Nephrology, Department of Internal Medicine, Kyung Hee University College of Medicine, Kyung Hee University Hospital, 23, Kyungheedae-ro, Dongdaemun-gu, Seoul, 02447 Republic of Korea; 2 Department of Pediatrics, Center for Digital Health, Medical Science Research Institute, Kyung Hee University College of Medicine, 23 Kyungheedae-ro, Dongdaemun-gu, Seoul, 02447 Republic of Korea; 3 Department of Medicine, University of Cambridge, Cambridge, UK; 4 Research and Development Unit, Parc Sanitari Sant Joan de Deu, CIBERSAM, ISCIII, Barcelona, Spain; 5 Catalan Institution for Research and Advanced Studies (ICREA), Pg. Lluis Companys, Barcelona, Spain; 6 Centre for Health, Performance and Wellbeing, Anglia Ruskin University, Cambridge, UK; 7 Department of Pediatrics, Severance Hospital, Yonsei University College of Medicine, Seoul, Republic of Korea; 8 Department of Data Science, Sejong University College of Software Convergence, Seoul, South Korea; 9 Department of Precision Medicine, Sungkyunkwan University School of Medicine, Suwon, South Korea

**Keywords:** Health care, Nephrology

## Abstract

Little is known about the prevalence of chronic kidney disease (CKD) during the coronavirus disease 2019 (COVID-19) pandemic. We aimed to investigate the long-term trends in CKD prevalence from South Korea including the early pandemic. We used data from 108,152 Korean adults from 2007 to 2020 obtained from a representative longitudinal serial study. We defined CKD as a condition when the participant’s estimated glomerular filtration rate was < 60 mL/min/1.73 m^2^, or one-time spot proteinuria was ≥ 1 +, and then examined the overall trends in the prevalence of CKD. Among the included adults (n = 80,010), the overall national prevalence of CKD was 6.2%. The trend slope gradually increased from 2007 to 2019, however, there was a sudden decrease in 2020 (2007–2010, 5.1% [95% confidence interval (CI) 4.7–5.5]; 2017–2019, 7.1% [95% CI 6.6–7.6]; pandemic period, 6.5% [95% CI 5.7–7.3]; and β_diff_, − 0.19; 95% CI − 0.24 to − 0.13). The prevalence of CKD among younger adults and those with poor medical utilization significantly decreased during the early pandemic. This study was the first large-scale study to investigate the longitudinal prevalence of CKD from 2007 to 2020. Further research is needed to fully understand the exact causes for this decline and to identify healthcare policy strategies for preventing and managing CKD.

## Introduction

The coronavirus disease 2019 (COVID-19) pandemic has significantly impacted socioeconomic conditions worldwide^1^. However, it is unknown whether the pandemic has had an impact on the prevalence of chronic kidney disease (CKD) in the general population^2^. Importantly, the belief that in-person outpatient clinic visits propagated the spread of COVID-19 had an impact on the number of outpatient clinic visits^3^. As the COVID-19 pandemic still has an impact on healthcare worldwide, identification of disadvantaged populations potentially limiting access to the best medical treatment is needed.

Chronic kidney disease (CKD) is a long-term condition associated with a high risk of death and is also connected to excess healthcare expenditure at advanced stages, and can ultimately require renal replacement therapy (RRT)^4^. CKD is a global public health problem, affecting 6.8–14.4% of the population across different countries^5^. The prevalence of CKD is known to increase with increasing prevalence of hypertension, diabetes, and obesity^6,7^. Unfortunately, CKD is clinically silent and asymptomatic until the later stages, and thus, many patients unaware of CKD prior to end stage of the condition^8,9^. Therefore, optimal screening via hospitals or workplaces and evidence-based management are critical to attenuate CKD progression and lower associated risks of end-stage kidney disease (ESKD) and mortality^8,10^. However, there is a lack of research on the prevalence of CKD during the COVID-19 pandemic. Therefore, it is necessary to conduct a long-term trend analysis based on the differences in CKD observed over before and during early stages of the COVID-19 pandemic.

Thus, the aim of this study was to evaluate the long-term nationwide prevalence of CKD among different subgroups including sociodemographic information, health-related lifestyle, and various medical conditions using nationally representative data from South Korea. Additionally, we assessed pandemic-related changes in the prevalence of CKD to examine whether the estimated prevalence of CKD in the early period of the pandemic differed from the expected level by comparing trends in the prevalence of the disease during the pre-pandemic (2007–2019) and early pandemic periods (2020).

## Methods

### Study population and design

This study utilized data from the Korea National Health and Nutrition Examination Survey (KNHANES) spanning from 2007 to 2020. KNHANES is a population-based nationwide longitudinal serial study that follows a multistage clustered design based on a stratified, multistage probability sampling scheme conducted by the Korea Disease Control and Prevention Agency (KDCA) for public interest^11,12^. In 2007, the frequency of the KNHANES has been modified from once every three years to every year in order to provide more timely statistics and diminish seasonal differences. Health interviews and examinations were conducted over 3 days in 192 primary sampling units across the country using the mobile examination center, consisting of two KNHANES-exclusive trucks^12^. Trained investigators performed all health examinations using validated methods and instruments calibrated periodically. This study was conducted in compliance with the ethical standards of relevant national and institutional committees on human experimentation and with the Helsinki Declaration of 1975, as revised in 2008. All procedures involving human subjects were approved by both the KDCA and Sejong University (SJU-HR-E-2020–003) institutional review board, and all anonymous participants signed a written informed consent form.

### Assessment of covariates and ascertainment of CKD

Sociodemographic information (age, sex, region, education, household income, and body mass index [BMI]), health-related lifestyle (smoking status), and various medical conditions were queried or self-reported. BMI (kg/m^2^) was calculated by dividing body weight (kg) by the square of height (m), and laboratory test results from blood and urine samples, including kidney function, were collected. The laboratory result data quality control program monitored the performance of all analytical values to meet the standard target of accuracy^12^. Medical conditions were applicable when participants self-reported diabetes mellitus, hypertension, dyslipidemia, myocardial infarction or ischemic heart disease, gastrointestinal malignancies, lung cancer, breast cancer, or uterine and cervical cancers. We classified residential areas into large cities and rural areas, including small- and medium-sized cities^11,13^. Educational level was divided into three categories: middle school or lower, high school, and college or higher^14^. Household income was categorized into quartiles: low, lower-middle, higher-middle, and high income. BMI was subdivided into three categories: underweight or normal BMI (< 23.0 kg/m^2^), overweight (23.0–25.0 kg/m^2^), and obese (≥ 25.0 kg/m^2^) with respect to Asia–Pacific BMI^15,16^. Current smoking was defined as smoking at least once within the last 30 days. Kidney function was assessed via estimated glomerular filtration rate (eGFR), calculated using the Chronic Kidney Disease Epidemiology Collaboration equation^17^. We defined CKD as a condition when the eGFR of the participant was lower than 60 mL/min/1.73 m^2^, one-time spot proteinuria was ≥ 1 + on a urine dipstick test according to Kidney Disease: Improving Global Outcomes practice guidelines^18^.

### Statistical analyses

KNHANES data analysis was performed between 2007 and 2020 following the protocol for a clustered, multi-stage, stratified sampling design to ensure a representative sample in Korea^11^. At first, we examined the overall trends in CKD during the study period (2007–2020) along with the age-standardized prevalence in overall CKD. The pre-pandemic period of the KNHANES cycle was set for four consecutive periods (2007–2010, 2011–2013, 2014–2016, and 2017–2019) to stabilize the data^19^. All analyses were stratified for the following baseline covariates: age, sex, residential area (urban and rural), educational level (middle school or lower, high school, and college or higher), household income (low, lower-middle, higher-middle, and high), BMI group (underweight or normal BMI, overweight, and obese), current smoking, outpatient clinic use within two weeks of the time of the survey, and medical condition (diabetes mellitus, hypertension, dyslipidemia, myocardial infarction or ischemic heart disease, gastrointestinal malignancies, lung cancer, breast cancer, or uterine and cervical cancer).

The baseline characteristics of the study participants were analyzed as weighted means with 95% confidence intervals (CIs) and frequencies with weighted proportions. We conducted a weighted complex sampling analysis using linear and logistic regression models. The trend difference was derived from the difference between β-coefficients before and after the pandemic to understand the trend of change in the prevalence of CKD during the entire period (2007–2020). The odds ratios (ORs) and 95% confidence intervals (CIs) were calculated using logistic regression models comparing the prevalence of CKD from 2007 to 2019 with 2020. Our next endpoint was whether the trends in the prevalence of CKD differed between the population who visited the outpatient clinic and those who did not visit the in-person clinic during the early COVID-19 pandemic era. All analyses were performed using SAS version 9.4 (SAS Inc., Cary, NC, USA)^20,21^. Statistical significance was defined as a two-sided *P*-value less than 0.05.

## Results

Of the subjects participating in the KNHANES 2007–2020 (total N = 108,152), we excluded those who (1) were under 19 years of age (excluded N = 23,889); (2) had missing data on covariates (e.g., household income) included in the multivariable model (excluded N = 909); and (3) did not have data for CKD ascertainment based on eGFR or proteinuria (excluded N = 3344). Thus, the final number of subjects for the analysis was 80,010.

Table 1 shows the general characteristics of the study participants. CKD accounted for 6.2% (unweighted n = 6105) adults of the total subjects, with 51.8% being females. A total of 50.0% (95% CI 48.3–51.6) of CKD group was 65 years or older, compared to 12.8% (95% CI 12.4–13.2) of the non-CKD group. The proportion of subjects with CKD in rural areas was higher than in those without CKD. The lower educational attainment group and the low quartile of household income group with CKD showed a higher proportion than those without CKD.

**Table 1:** Demographic characteristics of participating adults in the KNHANES, 2007–2020 (n = 80,010).

Characteristic	Non-CKD, n (%) or weighted % (95% CI)	CKD, n (%) or weighted % (95% CI)	Trends in prevalence of CKD				
			2007 to 2010	2011 to 2013	2014 to 2016	2017 to 2019	2020 (COVID-19 pandemic)
Number, n (%)	73,905 (93.8)	6105 (6.2)	1446 (5.1)	1180 (5.4)	1484 (7.1)	1538 (7.1)	457 (6.5)
Age, years, weighted mean (95% CI)							
19 ≤ Age < 65	87.2 (86.8 to 87.6)	50.0 (48.4 to 51.7)	50.8 (47.7 to 54.0)	43.9 (40.2 to 47.7)	49.8 (46.5 to 53.2)	53.9 (50.5 to 57.2)	49.4 (43.4 to 55.5)
Age ≥ 65	12.8 (12.4 to 13.2)	50.0 (48.3 to 51.6)	49.2 (46.0 to 52.3)	56.1 (52.3 to 59.8)	50.2 (46.8 to 53.5)	46.1 (42.8 to 49.5)	50.6 (44.5 to 56.6)
Sex, weighted % (95% CI)							
Male	49.8 (49.5 to 50.2)	48.2 (46.7 to 49.8)	47.0 (43.7 to 50.3)	46.4 (42.9 to 50.0)	43.7 (40.8 to 46.6)	52.0 (48.9 to 55.1)	56.5 (51.5 to 61.4)
Female	50.2 (49.8 to 50.5)	51.8 (50.2 to 53.3)	53.0 (49.7 to 56.3)	53.6 (50.0 to 57.1)	56.3 (53.4 to 59.2)	48.0 (44.9 to 51.1)	43.5 (38.6 to 48.5)
Residential area, weighted % (95% CI)							
Urban	46.8 (45.8 to 47.9)	43.5 (41.5 to 45.4)	46.2 (42.0 to 50.5)	44.0 (38.4 to 49.5)	42.4 (37.8 to 49.5)	42.5 (38.0 to 47.0)	42.2 (33.9 to 50.5)
Rural	53.2 (52.1 to 54.2)	56.5 (54.6 to 58.5)	53.8 (49.5 to 58.0)	56.0 (50.5 to 61.6)	57.6 (53.1 to 62.2)	57.5 (53.0 to 62.0)	57.8 (49.5 to 66.1)
Educational level, weighted % (95% CI)							
Middle school or lower	25.0 (24.4 to 25.6)	52.5 (50.8 to 54.2)	59.4 (55.8 to 62.9)	57.0 (53.2 to 60.9)	54.4 (51.0 to 57.8)	43.8 (40.5 to 47.1)	48.8 (42.2 to 55.3)
High school	28.4 (27.9 to 29.0)	21.9 (20.6 to 23.2)	20.8 (18.0 to 23.5)	22.7 (19.6 to 25.8)	22.0 (19.3 to 24.6)	21.9 (19.5 to 24.2)	23.0 (18.2 to 27.8)
College or higher	46.6 (45.8 to 47.3)	25.6 (24.1 to 27.1)	19.9 (16.9 to 22.9)	20.2 (16.8 to 23.6)	23.7 (20.9 to 26.4)	34.3 (30.9 to 37.7)	28.2 (22.2 to 34.3)
Household income, weighted % (95% CI)							
Low	25.0 (24.4 to 25.6)	29.7 (28.1 to 31.3)	27.1 (24.2 to 30.0)	28.5 (24.4 to 32.6)	30.7 (27.3 to 34.2)	27.1 (24.2 to 30.0)	31.1 (25.5 to 36.7)
Lower-middle	25.2 (24.6 to 25.7)	23.9 (22.5 to 25.2)	23.2 (20.6 to 25.7)	25.1 (21.6 to 28.5)	24.0 (21.3 to 26.7)	24.2 (21.6 to 26.7)	21.6 (17.0 to 26.3)
Higher-middle	25.2 (24.7 to 25.7)	22.2 (20.9 to 23.5)	23.7 (21.2 to 26.2)	23.0 (19.8 to 26.2)	21.7 (19.1 to 24.3)	21.7 (19.1 to 24.3)	19.9 (15.5 to 24.2)
High	24.6 (23.9 to 25.3)	24.2 (22.8 to 25.7)	26.1 (23.0 to 29.2)	23.5 (20.1 to 26.9)	23.6 (20.7 to 26.5)	23.0 (20.1 to 25.9)	27.4 (21.6 to 33.2)
BMI, kg/m^2^, weighted % (95% CI)							
BMI < 23.0	43.9 (43.5 to 44.4)	39.5 (38.0 to 41.0)	37.7 (34.7 to 40.8)	42.7 (39.2 to 46.1)	39.0 (35.8 to 42.1)	39.6 (36.8 to 42.4)	37.8 (32.5 to 43.1)
23.0 ≤ BMI < 25.0	23.0 (22.6 to 23.3)	21.7 (20.5 to 23.0)	21.6 (19.1 to 24.1)	18.9 (16.2 to 21.6)	24.5 (21.7 to 27.2)	19.8 (17.3 to 22.2)	26.3 (21.3 to 31.3)
BMI ≥ 25.0	33.1 (32.7 to 33.6)	38.8 (37.3 to 40.3)	40.6 (37.5 to 43.8)	38.4 (34.9 to 42.0)	36.6 (33.5 to 39.6)	40.6 (37.7 to 43.5)	35.9 (31.0 to 40.9)
Current smoking, weighted % (95% CI)							
No	55.9 (55.5 to 56.4)	57.8 (56.2 to 59.3)	52.7 (49.5 to 55.8)	59.4 (56.0 to 62.9)	64.1 (61.2 to 66.9)	56.2 (53.0 to 59.5)	52.4 (47.8 to 57.0)
Yes	44.1 (43.6 to 44.5)	42.2 (40.7 to 43.8)	47.3 (44.2 to 50.5)	40.6 (37.1 to 44.0)	35.9 (33.1 to 38.8)	43.8 (40.5 to 47.0)	47.6 (43.0 to 52.2)
Outpatient visit, weighted % (95% CI)							
No	72.2 (71.8 to 72.6)	62.1 (60.6 to 63.6)	55.0 (52.0 to 57.9)	55.4 (51.9 to 58.8)	62.9 (59.9 to 65.9)	68.1 (65.3 to 71.0)	71.6 (66.0 to 77.3)
Yes	27.8 (27.4 to 28.2)	37.9 (36.4 to 39.4)	45.0 (42.1 to 48.0)	44.6 (41.2 to 48.1)	37.1 (34.1 to 40.1)	31.9 (29.0 to 34.7)	28.4 (22.7 to 34.0)
Medical condition, weighted % (95% CI)							
Without	73.7 (73.2 to 74.1)	39.9 (38.3 to 41.5)	40.5 (37.4 to 43.6)	38.4 (34.6 to 42.2)	45.2 (41.8 to 48.5)	38.2 (34.9 to 41.4)	31.4 (26.8 to 36.0)
With	26.3 (25.9 to 26.8)	60.1 (58.5 to 61.7)	59.5 (56.4 to 62.6)	61.6 (57.8 to 65.4)	54.8 (51.5 to 58.2)	61.8 (58.6 to 65.1)	68.6 (64.0 to 73.2)

*BMI* body mass index, *CKD* chronic kidney disease, *CI* confidence interval, *KNHANES* Korea National Health and Nutrition Examination Survey.

### Trends of prevalence in patients with CKD

**Figure 1:**
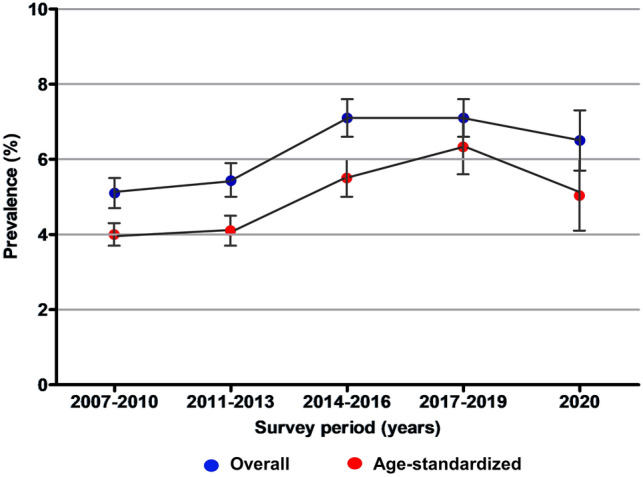
Nationwide 14-year trends and prevalence of CKD among Korean adults, 2007–2020 (n = 80,010).

The prevalence of CKD in adults was examined using KNHANES data over 14 years in subgroups according to sex, area of residence, education level, household income, BMI, current smoking status, outpatient clinic use, and medical conditions. The slope gradually increased from 2007 to 2019; however, there was a sudden decrease in 2020, as shown in Fig. 1. The weighted prevalence of CKD increased from 5.1% (95% CI 4.7–5.5) in 2007–2010 to 7.1% (95% CI 6.6–7.6) from 2017 to 2019, and then decreased to 6.5% (95% CI 5.7–7.3) in 2020 (Table 2). Similarly, the age-standardized prevalence of CKD increased from 4.0% (95% CI 3.7–4.3) in 2007–2010 to 6.3% (95% CI 5.6–7.1) from 2017 to 2019, and then decreased to 5.0% (95% CI 4.1–6.0) in 2020 (Table 2). The downward slope during the early COVID-19 period presented a consistent tendency in subgroups by age (19–64 years [β_diff_, − 0.27; 95% CI − 0.34 to − 0.19]), sex (male [β_diff_, − 0.20; 95% CI − 0.28 to − 0.13] and female [β_diff_, − 0.18; 95% CI − 0.25 to − 0.11]), residential area (urban [β_diff_, − 0.15; 95% CI − 0.24 to − 0.07] and rural [β_diff_, − 0.21; 95% CI − 0.29 to − 0.14]), educational attainment (middle school or lower [β_diff_, − 0.09; 95% CI − 0.16 to − 0.02], high school [β_diff_, − 0.27; 95% CI − 0.37 to − 0.17], and college or higher [β_diff_, − 0.39; 95% CI − 0.50 to − 0.29]), household income (low [β_diff_, − 0.24; 95% CI − 0.34 to − 0.15], lower-middle [β_diff_, − 0.22; 95% CI − 0.32 to − 0.11], higher-middle [β_diff_, − 0.17; 95% CI − 0.28 to − 0.07], and high [β_diff_, − 0.10; 95% CI − 0.21–0.00, *P* < 0.05]), BMI group (underweight or normal [β_diff_, − 0.20; 95% CI − 0.28 to − 0.12], overweight [β_diff_, − 0.13; 95% CI − 0.23 to − 0.03], and obese [β_diff_, − 0.20; 95% CI − 0.29 to − 0.12]), current smoking status (no [β_diff_, − 0.20; 95% CI − 0.26 to − 0.13], yes [β_diff_, − 0.17; 95% CI − 0.25 to − 0.09]), and medical conditions (those without [β_diff_, − 0.26; 95% CI − 0.34 to − 0.18], those with [β_diff_, − 0.07; 95% CI − 0.13–0.00, *P* < 0.05]).

**Table 2:** Chronic kidney disease weighted prevalence and trend in adults from 2007–2020.

	Trends in prevalence of CKD								
	2007 to 2010	2011 to 2013	2014 to 2016	2017 to 2019	2020 (early pandemic)	Trend in the pre-pandemic, β (95% CI)^a^	Trend in the early pandemic, β (95% CI)^a^	Trend difference, β_diff_ (95% CI)	OR (95% CI)^b^
CKD, weighted % (95% CI)									
Overall	5.1 (4.7 to 5.5)	5.4 (5.0 to 5.9)	7.1 (6.6 to 7.6)	7.1 (6.6 to 7.6)	6.5 (5.7 to 7.3)	**0.16 (0.12 to 0.20)**	− 0.02 (− 0.06 to 0.01)	− **0.19 (**− **0.24 to **− **0.13)**	0.92 (0.81 to 1.05)
Age-standardized	4.0 (3.7 to 4.3)	4.1 (3.7 to 4.5)	5.5 (5.0 to 6.0)	6.3 (5.6 to 7.1)	5.0 (4.1 to 6.0)	**0.17 (0.14 to 0.20)**	− 0.04 (− 0.08 to 0.00)	− **0.21 (**− **0.26 to **− **0.16)**	0.95 (0.81 to 1.06)
Age									
19 ≤ Age < 65	3.0 (2.7 to 3.3)	2.8 (2.4 to 3.1)	4.2 (3.7 to 4.6)	4.6 (4.2 to 5.1)	4.0 (3.2 to 4.7)	**0.23 (0.17 to 0.28)**	− 0.04 (− 0.09 to 0.01)	− **0.27 (**− **0.34 to **− **0.19)**	1.04 (0.85 to 1.27)
Age ≥ 65	19.7 (18.2 to 21.3)	21.7 (20.0 to 23.5)	22.9 (21.3 to 24.6)	19.3 (17.8 to 20.8)	17.9 (15.8 to 20.0)	− 0.01 (− 0.06 to 0.04)	− 0.02 (− 0.07 to 0.02)	− 0.01 (− 0.08 to 0.06)	**0.74 (0.63 to 0.85)**
Sex									
Male	4.8 (4.3 to 5.3)	4.2 (3.7 to 4.6)	6.3 (5.7 to 6.9)	7.4 (6.8 to 8.1)	7.4 (6.2 to 8.6)	**0.20 (0.15 to 0.26)**	0.00 (− 0.05 to 0.05)	− **0.20 (**− **0.28 to **− **0.13)**	1.11 (0.93 to 1.32)
Female	5.4 (4.9 to 5.9)	5.8 (5.1 to 6.4)	7.9 (7.2 to 8.6)	6.8 (6.2 to 7.4)	5.6 (4.8 to 6.5)	**0.13 (0.07 to 0.18)**	− 0.05 (− 0.10 to 0.00)	− **0.18 (**− **0.25 to **− **0.11)**	**0.75 (0.63 to 0.89)**
Residential area									
Urban	5.0 (4.5 to 5.5)	5.1 (4.4 to 5.8)	6.4 (5.7 to 7.2)	6.6 (5.9 to 7.2)	6.2 (5.0 to 7.4)	**0.14 (0.08 to 0.20)**	− 0.01 (− 0.07 to 0.05)	− **0.15 (**− **0.24 to **− **0.07)**	0.92 (0.75 to 1.14)
Rural	5.2 (4.7 to 5.7)	5.7 (5.1 to 6.4)	7.7 (7.0 to 8.4)	7.6 (6.9 to 8.3)	6.8 (5.7 to 7.8)	**0.18 (0.13 to 0.24)**	− 0.03 (− 0.08 to 0.02)	− **0.21 (**− **0.29 to **− **0.14)**	0.93 (0.79 to 1.09)
Educational level									
Middle school or lower	10.7 (10.7 to 10.7)	11.4 (11.4 to 11.4)	13.6 (13.6 to 13.6)	12.9 (12.9 to 12.9)	13.7 (13.7 to 13.7)	**0.10 (0.05 to 0.15)**	0.02 (− 0.03 to 0.07)	− **0.09 (**− **0.16 to **− **0.02)**	0.89 (0.74 to 1.06)
High school	3.5 (3.0 to 4.0)	4.1 (3.5 to 4.7)	6.0 (5.1 to 6.8)	6.0 (5.3 to 6.7)	5.7 (4.3 to 7.0)	**0.25 (0.18 to 0.33)**	− 0.01 (− 0.09 to 0.06)	− **0.27 (**− **0.37 to **− **0.17)**	0.87 (0.66 to 1.15)
College or higher	2.5 (2.1 to 2.9)	2.6 (2.1 to 3.1)	3.7 (3.2 to 4.2)	4.9 (4.3 to 5.5)	3.7 (2.8 to 4.5)	**0.32 (0.24 to 0.40)**	− **0.08 **− **(0.14 to **− **0.01)**	− **0.39 (**− **0.50 to **− **0.29)**	0.91 (0.71 to 1.17)
Household income									
Low	5.5 (4.8 to 6.2)	5.9 (4.9 to 7.0)	8.6 (7.5 to 9.8)	8.8 (7.9 to 9.7)	8.3 (6.7 to 10.0)	**0.23 (0.16 to 0.30)**	− 0.01 (− 0.08 to 0.05)	− **0.24 (**− **0.34 to **− **0.15)**	0.96 (0.77 to 1.20)
Lower-middle	4.8 (4.2 to 5.4)	5.3 (4.5 to 6.2)	6.8 (5.9 to 7.7)	6.8 (6.0 to 7.7)	5.7 (4.4 to 7.1)	**0.17 (0.10 to 0.24)**	− 0.05 (− 0.12 to 0.03)	− **0.22 (**− **0.32 to **− **0.11)**	0.84 (0.64 to 1.11)
Higher-middle	4.8 (4.2 to 5.4)	5.0 (4.2 to 5.8)	6.2 (5.4 to 7.1)	6.1 (5.3 to 7.0)	5.1 (3.9 to 6.3)	**0.12 (0.04 to 0.20)**	− 0.05 (− 0.12 to 0.02)	− **0.17 (**− **0.28 to **− **0.07)**	0.80 (0.62 to 1.04)
High	5.3 (4.6 to 6.0)	5.4 (4.6 to 6.2)	6.7 (5.8 to 7.6)	6.7 (5.8 to 7.6)	7.0 (5.6 to 8.4)	**0.12 (0.04 to 0.20)**	0.01 (− 0.05 to 0.08)	− **0.10 (**− **0.21 to 0.00)**	1.04 (0.82 to 1.32)
BMI									
BMI < 23.0	4.3 (3.8 to 4.8)	5.2 (4.6 to 5.8)	6.4 (5.7 to 7.1)	6.6 (5.9 to 7.2)	6.3 (5.1 to 7.5)	**0.19 (0.13 to 0.25)**	− 0.01 (− 0.07 to 0.05)	− **0.20 (**− **0.28 to **− **0.12)**	0.93 (0.76 to 1.14)
23.0 ≤ BMI < 25.0	4.7 (4.1 to 5.3)	4.5 (3.8 to 5.2)	7.5 (6.5 to 8.5)	6.3 (5.5 to 7.1)	7.5 (5.9 to 9.1)	**0.18 (0.10 to 0.25)**	0.05 (− 0.02 to 0.11)	− **0.13 (**− **0.23 to **− **0.03)**	1.21 (0.93 to 1.58)
BMI ≥ 25.0	6.6 (5.9 to 7.3)	6.4 (5.6 to 7.2)	7.7 (6.9 to 8.5)	8.3 (7.5 to 9.2)	6.2 (5.1 to 7.3)	**0.12 (0.06 to 0.19)**	− **0.08 (**− **0.13 to **− **0.03)**	− **0.20 (**− **0.29 to **− **0.12)**	**0.77 (0.63 to 0.94)**
Current smoking									
No	5.1 (4.6 to 5.5)	5.8 (5.1 to 6.4)	7.8 (7.2 to 8.5)	7.0 (6.4 to 7.6)	6.0 (5.1 to 6.8)	**0.15 (0.10 to 0.20)**	− **0.04 (**− **0.09 to 0.00)**	− **0.20 (**− **0.26 to **− **0.13)**	**0.80 (0.68 to 0.94)**
Yes	5.1 (4.6 to 5.7)	5.0 (4.4 to 5.6)	6.1 (5.4 to 6.7)	7.3 (6.6 to 8.0)	7.3 (6.1 to 8.4)	**0.17 (0.11 to 0.23)**	0.00 (− 0.05 to 0.05)	− **0.17 (**− **0.25 to **− **0.09)**	1.09 (0.91 to 1.31)
Outpatient visit									
No	4.0 (3.6 to 4.3)	4.3 (3.8 to 4.8)	6.2 (5.7 to 6.8)	6.7 (6.2 to 7.2)	6.2 (5.4 to 7.1)	**0.25 (0.20 to 0.30)**	− 0.02 (− 0.06 to 0.02)	− **0.27 (**− **0.33 to **− **0.20)**	0.97 (0.83 to 1.13)
Yes	7.9 (7.2 to 8.6)	8.1 (7.2 to 8.9)	9.3 (8.4 to 10.2)	8.2 (7.3 to 9.1)	7.5 (5.8 to 9.2)	0.04 − (0.02 to 0.10)	− 0.03 (− 0.09 to 0.04)	− 0.06 (− 0.15 to 0.03)	0.83 (0.66 to 1.05)
Medical condition									
Without	2.8 (2.5 to 3.0)	2.8 (2.4 to 3.2)	4.5 (4.0 to 5.0)	4.0 (3.6 to 4.4)	3.1 (2.5 to 3.8)	**0.20 (0.14 to 0.26)**	− **0.06 (**− **0.12 to **− **0.01)**	− **0.26 (**− **0.34 to **− **0.18)**	0.90 (0.73 to 1.12)
With	12.3 (11.2 to 13.3)	13.0 (11.9 to 14.1)	13.6 (12.5 to 14.7)	13.7 (12.6 to 14.7)	12.8 (11.3 to 14.3)	**0.05 (0.00 to 0.10)**	− 0.02 (− 0.06 to 0.02)	− **0.07 (**− **0.13 to 0.00)**	0.89 (0.77 to 1.03)

*BMI* body mass index, *CKD* chronic kidney disease, *CI* confidence interval, *KNHANES* Korea National Health and Nutrition Examination Survey, *OR* odds ratio.

^a^Estimated β was calculated to analyze the year cycle (2007–2010, 2011–2013, 2014–2016, 2017–2019, and 2020) as a continuous variable using a linear regression model.

^b^Estimated OR was calculated to analyze the year cycle (2017–2019 versus 2020 [COVID-19 pandemic]) as a categorical variable using a logistic regression model.

The numbers in bold indicate a significant difference (*p* < 0.05).

Although we compared trends before and during the early stage of COVID-19 pandemic, there was no significant difference in the slopes of the older adult and outpatient clinic visit groups. The odds of CKD in females decreased during the early COVID-19 pandemic from 2007 to 2019 (OR 0.75; 95% CI 0.63–0.89).

### Differences in prevalence trends in patients with chronic kidney disease according to outpatient clinic visits

The prevalence of CKD in participants who did not visit the outpatient clinic within 2 weeks is presented in Table 3. Similar to the results in Table 2, the prevalence of participants without outpatient clinic visits increased until 2019 but stagnated in 2020 in most subgroups. In Table 3, the overall β values were positive in the pre-pandemic period and neutral in the early pandemic period, representing a statistically significant decrement in the trend difference of prevalence (β_diff_, − 0.27; 95% CI − 0.33 to − 0.20). The increasing trend in the prevalence of CKD during the pre-pandemic period was consistent regardless of sex, residential area, educational attainment, household income, BMI group, current smoking status, and medical condition. Older adults showed no significant change in β both prior to and during the COVID-19 pandemic. Diminishing trends were observed in certain subgroups of participants during the early pandemic period: age 19 to 64 years, college or higher, and BMI ≥ 25.0 kg/m^2^. The β_diff_ in the prevalence of CKD in all subgroups without outpatient clinic utilization, except for older adults, were negative.

**Table 3:** Chronic kidney disease weighted prevalence and trend in adults who did not visit outpatient clinic within 2 weeks from 2007–2020.

	Trends in prevalence of CKD								
	2007 to 2010	2011 to 2013	2014 to 2016	2017 to 2019	2020 (COVID-19 pandemic)	Trend in the pre-pandemic, β (95% CI)^a^	Trend in the early pandemic, β (95% CI)^a^	Trend difference, β_diff_ (95% CI)	OR (95% CI)^b^
CKD, weighted % (95% CI)									
Overall	4.0 (3.6 to 4.3)	4.3 (3.8 to 4.8)	6.2 (5.7 to 6.8)	6.7 (6.2 to 7.2)	6.2 (5.4 to 7.1)	**0.25 (0.20 to 0.30)**	− 0.02 (− 0.06 to 0.02)	− **0.27 (**− **0.33 to **− **0.20)**	0.97 (0.83 to 1.13)
Age									
19 ≤ Age < 65	2.5 (2.1 to 2.8)	2.5 (2.1 to 2.8)	3.9 (3.4 to 4.4)	4.6 (4.1 to 5.1)	3.6 (2.9 to 4.3)	**0.31 (0.24 to 0.37)**	− **0.06 (**− **0.12 to 0.00)**	− **0.37 (**− **0.46 to **− **0.28)**	1.01 (0.80 to 1.27)
Age ≥ 65	18.8 (16.8 to 20.8)	21.0 (18.7 to 23.4)	22.8 (20.7 to 25.0)	19.2 (17.4 to 21.0)	19.3 (16.5 to 22.0)	0.01 (− 0.06 to 0.07)	0.00 (− 0.05 to 0.05)	− 0.01 (− 0.09 to 0.08)	**0.80 (0.66 to 0.97)**
Sex									
Male	3.8 (3.3 to 4.3)	4.1 (3.5 to 4.7)	5.5 (4.8 to 6.1)	6.8 (6.1 to 7.6)	6.4 (5.3 to 7.6)	**0.28 (0.21 to 0.35)**	− 0.02 (− 0.07 to 0.04)	− **0.29 (**− **0.38 to **− **0.20)**	1.06 (0.86 to 1.30)
Female	4.2 (3.7 to 4.7)	4.5 (3.9 to 5.2)	7.0 (6.2 to 7.8)	6.5 (5.8 to 7.2)	6.0 (4.9 to 7.0)	**0.22 (0.16 to 0.28)**	− 0.02 (− 0.08 to 0.03)	− **0.24 (**− **0.33 to **− **0.16)**	0.87 (0.71 to 1.07)
Residential area									
Urban	3.8 (3.3 to 4.4)	4.0 (3.4 to 4.7)	5.6 (4.9 to 6.4)	6.1 (5.3 to 6.8)	6.0 (4.7 to 7.3)	**0.22 (0.15 to 0.30)**	0.00 (− 0.07 to 0.06)	− **0.23 (**− **0.33 to **− **0.13)**	0.97 (0.76 to 1.25)
Rural	4.1 (3.6 to 4.6)	4.5 (3.8 to 5.2)	6.7 (5.9 to 7.5)	7.2 (6.5 to 8.0)	6.4 (5.3 to 7.5)	**0.27 (0.20 to 0.33)**	− 0.03 (− 0.09 to 0.02)	− **0.30 (**− **0.39 to **− **0.22)**	0.96 (0.78 to 1.18)
Educational level									
Middle school or lower	9.1 (8.1 to 10.0)	9.3 (8.1 to 10.6)	12.6 (11.3 to 13.9)	11.9 (10.7 to 13.1)	14.0 (11.5 to 16.5)	**0.15 (0.09 to 0.21)**	0.05 (− 0.01 to 0.10)	− **0.10 (**− **0.19 to **− **0.02)**	1.01 (0.81 to 1.27)
High school	3.0 (2.4 to 3.5)	3.5 (2.8 to 4.2)	5.1 (4.1 to 6.0)	6.0 (5.1 to 6.9)	4.7 (3.2 to 6.2)	**0.32 (0.22 to 0.41)**	− 0.06 (− 0.15 to 0.03)	− **0.38 (**− **0.51 to **− **0.25)**	0.79 (0.54 to 1.14)
College or higher	2.0 (1.6 to 2.4)	2.3 (1.7 to 2.8)	3.2 (2.6 to 3.8)	4.8 (4.1 to 5.4)	3.4 (2.5 to 4.3)	**0.39 (0.30 to 0.49)**	− **0.09 (**− **0.16 to **− **0.01)**	− **0.48 (**− **0.60 to **− **0.36)**	0.95 (0.71 to 1.27)
Household income									
Low	4.3 (3.6 to 5.1)	4.8 (3.7 to 5.8)	7.8 (6.5 to 9.0)	8.2 (7.2 to 9.2)	7.6 (5.8 to 9.3)	**0.30 (0.22 to 0.39)**	− 0.02 (− 0.09 to 0.05)	− **0.33 (**− **0.44 to **− **0.21)**	0.95 (0.73 to 1.24)
Lower-middle	3.6 (2.9 to 4.2)	4.0 (3.1 to 4.9)	5.9 (4.9 to 6.8)	6.3 (5.4 to 7.2)	4.9 (3.4 to 6.4)	**0.26 (0.17 to 0.36)**	− 0.07 (− 0.16 to 0.02)	− **0.33 (**− **0.46 to **− **0.20)**	0.79 (0.55 to 1.13)
Higher-middle	3.8 (3.1 to 4.4)	4.0 (3.2 to 4.8)	5.6 (4.6 to 6.6)	6.0 (5.0 to 7.0)	4.7 (3.4 to 6.1)	**0.22 (0.12 to 0.32)**	− 0.06 (− 0.15 to 0.03)	− **0.28 (**− **0.42 to **− **0.15)**	0.79 (0.57 to 1.09)
High	4.2 (3.5 to 4.9)	4.4 (3.6 to 5.3)	5.7 (4.7 to 6.6)	6.3 (5.3 to 7.3)	7.6 (5.8 to 9.5)	**0.19 (0.10 to 0.29)**	0.05 (− 0.03 to 0.13)	− **0.15 (**− **0.27 to **− **0.02)**	1.30 (0.98 to 1.73)
BMI									
BMI < 23.0	3.2 (2.8 to 3.7)	4.0 (3.4 to 4.6)	5.6 (4.9 to 6.3)	6.2 (5.5 to 6.9)	6.5 (5.0 to 8.0)	**0.29 (0.22 to 0.36)**	0.01 (− 0.06 to 0.08)	− **0.27 (**− **0.37 to **− **0.18)**	1.08 (0.83 to 1.40)
23.0 ≤ BMI < 25.0	3.6 (2.9 to 4.2)	3.8 (3.0 to 4.7)	6.7 (5.5 to 7.8)	5.8 (4.8 to 6.8)	6.8 (5.1 to 8.6)	**0.25 (0.15 to 0.34)**	0.04 (− 0.04 to 0.12)	− **0.20 (**− **0.33 to **− **0.08)**	1.20 (0.87 to 1.66)
BMI ≥ 25.0	5.4 (4.6 to 6.1)	5.0 (4.1 to 5.8)	6.8 (5.9 to 7.7)	7.8 (6.9 to 8.7)	5.5 (4.4 to 6.7)	**0.20 (0.12 to 0.28)**	− **0.09 (**− **0.16 to **− **0.03)**	− **0.29 (**− **0.39 to **− **0.19)**	**0.75 (0.59 to 0.95)**
Current smoking									
No	4.0 (3.5 to 4.5)	4.8 (4.2 to 5.4)	6.8 (6.1 to 7.6)	6.7 (6.0 to 7.4)	6.4 (5.4 to 7.5)	**0.23 (0.17 to 0.29)**	− 0.01 (− 0.06 to 0.04)	− **0.24 (**− **0.32 to **− **0.16)**	0.97 (0.80 to 1.17)
Yes	4.0 (3.5 to 4.5)	3.7 (3.1 to 4.3)	5.5 (4.8 to 6.2)	6.7 (5.9 to 7.5)	5.9 (4.7 to 7.1)	**0.27 (0.20 to 0.35)**	− 0.03 (− 0.10 to 0.03)	− **0.30 (**− **0.40 to **− **0.21)**	0.95 (0.76 to 1.19)
Medical condition									
Without	2.3 (2.0 to 2.7)	2.6 (2.2 to 3.0)	4.4 (3.9 to 5.0)	4.1 (3.6 to 4.6)	3.3 (2.6 to 4.0)	**0.27 (0.20 to 0.34)**	− 0.05 (− 0.12 to 0.01)	− **0.32 (**− **0.41 to **− **0.23)**	0.98 (0.77 to 1.25)
With	11.0 (9.7 to 12.2)	11.4 (10.0 to 12.8)	12.0 (10.7 to 13.3)	13.4 (12.1 to 14.7)	12.6 (10.8 to 14.4)	**0.10 (0.03 to 0.17)**	− 0.02 (− 0.07 to 0.03)	− **0.12 (**− **0.20 to **− **0.03)**	0.88 (0.73 to 1.06)

*BMI* body mass index, *CKD* chronic kidney disease, *CI* confidence interval, *KNHANES* Korea National Health and Nutrition Examination Survey, *OR* odds ratio.

^a^Estimated β was calculated to analyze the year cycle (2007–2010, 2011–2013, 2014–2016, 2017–2019, and 2020) as a continuous variable using a linear regression model.

^b^Estimated OR was calculated to analyze the year cycle (2017–2019 versus 2020 [COVID-19 pandemic]) as a categorical variable using a logistic regression model.

The numbers in bold indicate a significant difference (*p* < 0.05).

The trend of CKD prevalence in participants who used outpatient clinics within 2 weeks is presented in Table 4, including the prevalence for each subgroup. Overall, there was no significant difference in trend difference (β_diff_, − 0.06; 95% CI − 0.15–0.03) and odds ratio (OR 0.83; 95% CI 0.66–1.05) before and at the beginning of the COVID-19 period. 19.4% (95% CI 17.2–21.7) of older adults satisfied the criteria for CKD in 2017–2019, and then only 15.0% (95% CI 11.4–18.6) accounted the for CKD population in 2020 (β_diff_, − 0.08; 95% CI − 0.15–0.00; *P* < 0.05). β_diff_ were significantly negative in females (β_diff_, − 0.12; 95% CI − 0.23 to − 0.01) and patients without current smoking (β_diff_, − 0.18; 95% CI − 0.29 to − 0.07) with outpatient visits, respectively, and odds were also similar in females (OR 0.53; 95% CI 0.39–0.73) and those without current smoking (OR 0.47; 95% CI 0.34–0.65; Table 4). In participants with outpatient clinic use, the β of prevalence increased from 2007 to 2019 across educational levels, and a decline in β_diff_ was observed in those with middle school or lower (β_diff_, − 0.12; 95% CI − 0.23 to − 0.01) and college or higher (β_diff_, − 0.19; 95% CI − 0.38 to − 0.01), respectively. The odds for both high household income (OR 0.55; 95% CI 0.34–0.88) and underweight and normal weight (OR 0.64; 95% CI 0.45–0.91) were significantly low.

**Table 4:** Chronic kidney disease weighted prevalence and trend in adults who visited the outpatient clinic within 2 weeks from 2007–2020.

	Trends in prevalence of chronic kidney disease								
	2007 to 2010	2011 to 2013	2014 to 2016	2017 to 2019	2020 (early pandemic)	Trend in the pre-pandemic, β (95% CI)^a^	Trend in the early pandemic, β (95% CI)^a^	Trend difference, β_diff_ (95% CI)	OR (95% CI)^b^
CKD, weighted % (95% CI)									
Overall	7.9 (7.2 to 8.6)	8.1 (7.2 to 8.9)	9.3 (8.4 to 10.2)	8.2 (7.3 to 9.1)	7.5 (5.8 to 9.2)	0.04 (− 0.02 to 0.10)	− 0.03 (− 0.09 to 0.04)	− 0.06 (− 0.15 to 0.03)	0.83 (0.66 to 1.05)
Age									
19 ≤ Age < 65	4.4 (3.8 to 5.1)	3.6 (3.0 to 4.3)	5.1 (4.2 to 5.9)	4.6 (3.7 to 5.5)	5.1 (3.5 to 6.8)	0.06 (− 0.04 to 0.16)	0.03 (− 0.07 to 0.13)	− 0.03 (− 0.17 to 0.11)	1.12 (0.80 to 1.57)
Age ≥ 65	20.8 (18.7 to 22.8)	22.4 (20.1 to 24.8)	23.0 (20.7 to 25.4)	19.4 (17.2 to 21.7)	15.0 (11.4 to 18.6)	− 0.03 (− 0.10 to 0.04)	− **0.08 (**− **0.15 to 0.00)**	− 0.05 (− 0.15 to 0.06)	**0.62 (0.47 to 0.84)**
Sex									
Male	8.0 (6.8 to 9.2)	7.9 (6.7 to 9.1)	8.7 (7.4 to 10.1)	9.3 (7.9 to 10.6)	10.9 (7.9 to 13.9)	0.08 (− 0.02 to 0.17)	0.04 (− 0.04 to 0.13)	− 0.03 (− 0.16 to 0.10)	1.23 (0.92 to 1.66)
Female	7.8 (6.9 to 8.7)	8.2 (7.0 to 9.3)	9.8 (8.6 to 10.9)	7.5 (6.4 to 8.6)	4.8 (3.4 to 6.3)	0.01 (− 0.07 to 0.08)	− **0.11 (**− **0.20 to **− **0.03)**	− **0.12 (**− **0.23 to **− **0.01)**	**0.53 (0.39 to 0.73)**
Residential area									
Urban	7.8 (6.7 to 8.9)	7.6 (6.2 to 9.0)	8.4 (7.1 to 9.8)	7.9 (6.6 to 9.2)	6.9 (4.6 to 9.1)	0.02 (− 0.08 to 0.11)	− 0.04 (− 0.13 to 0.06)	− 0.05 (− 0.19 to 0.08)	0.82 (0.58 to 1.15)
Rural	7.9 (7.0 to 8.9)	8.4 (7.3 to 9.6)	10.2 (9.0 to 11.3)	8.6 (7.3 to 9.8)	7.9 (5.5 to 10.3)	0.052 (− 0.03 to 0.13)	− 0.02 (− 0.11 to 0.07)	− 0.07 (− 0.19 to 0.05)	0.85 (0.62 to 1.16)
Educational level									
Middle school or lower	13.0 (11.7 to 14.4)	14.2 (12.4 to 16.0)	15.7 (13.8 to 17.5)	15.0 (13.0 to 16.9)	12.9 (9.3 to 16.4)	**0.07 (0.00 to 0.15)**	− 0.04 (− 0.13 to 0.04)	− **0.12 (**− **0.23 to **− **0.01)**	**0.60 (0.43 to 0.83)**
High school	5.0 (3.9 to 6.1)	5.5 (4.3 to 6.7)	8.4 (6.6 to 10.1)	6.0 (4.7 to 7.4)	8.3 (5.4 to 11.3)	**0.12 (0.01 to 0.24)**	0.09 (− 0.03 to 0.20)	− 0.04 (− 0.20 to 0.12)	1.06 (0.71 to 1.59)
College or higher	4.1 (3.0 to 5.1)	3.5 (2.6 to 4.4)	5.0 (4.0 to 6.0)	5.3 (4.1 to 6.4)	4.4 (2.6 to 6.2)	**0.15 (0.01 to 0.28)**	− 0.05 (− 0.17 to 0.08)	− **0.19 (**− **0.38 to **− **0.01)**	0.84 (0.54 to 1.30)
Household income									
Low	8.5 (7.0 to 9.9)	8.5 (6.9 to 10.2)	10.8 (8.8 to 12.8)	10.4 (8.4 to 12.4)	10.9 (6.8 to 15.1)	**0.12 (0.01 to 0.23)**	0.01 (− 0.11 to 0.13)	− 0.10 (− 0.26 to 0.06)	0.99 (0.66 to 1.51)
Lower-middle	7.6 (6.3 to 8.9)	8.3 (6.7 to 10.0)	9.4 (7.6 to 11.2)	8.3 (6.7 to 9.9)	8.6 (5.6 to 11.7)	0.05 (− 0.06 to 0.16)	0.01 (− 0.10 to 0.12)	− 0.04 (− 0.19 to 0.12)	0.94 (0.64 to 1.39)
Higher-middle	7.4 (6.1 to 8.8)	7.5 (5.9 to 9.1)	7.8 (6.3 to 9.4)	6.6 (5.2 to 8.0)	6.0 (3.6 to 8.3)	− 0.04 (− 0.15 to 0.07)	− 0.03 (− 0.15 to 0.09)	0.01 (− 0.15 to 0.18)	0.82 (0.52 to 1.27)
High	8.0 (6.6 to 9.5)	7.7 (6.1 to 9.4)	9.2 (7.6 to 10.9)	7.6 (5.9 to 9.2)	5.1 (2.9 to 7.2)	0.00 (− 0.12 to 0.12)	− 0.10 (− 0.22 to 0.02)	− 0.10 (− 0.27 to 0.06)	**0.55 (0.34 to 0.88)**
BMI									
BMI < 23.0	7.1 (6.0 to 8.2)	8.0 (6.7 to 9.3)	8.6 (7.2 to 10.0)	7.5 (6.2 to 8.7)	5.7 (3.7 to 7.6)	0.03 (− 0.07 to 0.12)	− 0.07 (− 0.17 to 0.03)	− 0.10 (− 0.24 to 0.04)	**0.64 (0.45 to 0.91)**
23.0 ≤ BMI < 25.0	7.4 (6.0 to 8.7)	6.0 (4.7 to 7.2)	9.7 (7.9 to 11.6)	7.5 (6.0 to 9.0)	9.5 (6.1 to 12.8)	0.07 (− 0.04 to 0.19)	0.06 (− 0.05 to 0.18)	− 0.01 (− 0.17 to 0.15)	1.22 (0.79 to 1.88)
BMI ≥ 25.0	9.2 (8.0 to 10.5)	9.6 (8.1 to 11.2)	10.0 (8.6 to 11.4)	9.7 (8.0 to 11.3)	8.2 (5.6 to 10.8)	0.02 (− 0.07 to 0.12)	− 0.04 (− 0.14 to 0.05)	− 0.07 (− 0.20 to 0.07)	0.83 (0.58 to 1.19)
Current smoking									
No	7.4 (6.5 to 8.3)	7.9 (6.8 to 9.1)	10.3 (9.1 to 11.6)	7.8 (6.7 to 8.85)	4.7 (3.2 to 6.1)	0.05 (− 0.02 to 0.13)	− **0.13 (**− **0.21 to **− **0.05)**	− **0.18 (**− **0.29 to **− **0.07)**	**0.47 (0.34 to 0.65)**
Yes	8.6 (7.4 to 9.8)	8.2 (7.0 to 9.5)	7.8 (6.5 to 9.0)	9.0 (7.5 to 10.5)	11.5 (8.4 to 14.7)	0.01 (− 0.08 to 0.11)	0.07 (− 0.02 to 0.16)	0.06 (− 0.07 to 0.19)	1.48 (1.09 to 2.00)
Medical condition									
Without	2.3 (2.0 to 2.7)	2.6 (2.2 to 3.0)	4.4 (3.9 to 5.0)	4.1 (3.6 to 4.6)	2.4 (1.0 to 3.8)	0.01 (− 0.11 to 0.12)	− 0.11 (− 0.26 to 0.03)	− 0.12 (− 0.30 to 0.07)	0.60 (0.35 to 1.04)
With	11.0 (9.7 to 12.2)	11.4 (10.0 to 12.8)	12.0 (10.7 to 13.3)	13.4 (12.1 to 14.7)	13.2 (10.4 to 16.0)	0.02 (− 0.05 to 0.08)	− 0.02 (− 0.09 to 0.05)	− 0.03 (− 0.13 to 0.07)	0.89 (0.69 to 1.16)

*BMI* body mass index, *CKD* chronic kidney disease, *CI* confidence interval, *KNHANES* Korea National Health and Nutrition Examination Survey, *OR* odds ratio.

^a^Estimated β was calculated to analyze the year cycle (2007–2010, 2011–2013, 2014–2016, 2017–2019, and 2020) as a continuous variable using a linear regression model.

^b^Estimated OR was calculated to analyze the year cycle (2017–2019 versus 2020 [COVID-19 pandemic]) as a categorical variable using a logistic regression model.

The numbers in bold indicate a significant difference (*p* < 0.05).

## Discussion

This nationwide representative longitudinal serial study included a long-term trend analysis from 2007 to 2020. This is the first study to examine the 14-year trends in the prevalence of CKD, including the early period of COVID-19. Although the trends of both overall and age-standardized prevalence of CKD increased before the pandemic (2007–2019), the overall prevalence and age-standardized prevalence of CKD significantly decreased during the early pandemic (2020) in similar pattern. The overall trend of the prevalence of CKD in the early COVID-19 pandemic was not significantly different from that of CKD in the pre-pandemic period in older patients or patients who visited outpatient clinics. Interestingly, β_diff_ values of younger patients or those who did not visit the outpatient clinic were negative in the overall group.

To the best of our knowledge, this is the first study to investigate 14-year trends in the prevalence of CKD, including the early COVID-19 period, as a nationwide representative serial study. The increase in rates of overall prevalence of CKD became less steep before the outbreak of the COVID-19 pandemic in Japan, the UK, and the USA^22–24^. However, although the pre-pandemic prevalence of CKD in Korea was similar to that of the aforementioned countries, the prevalence of CKD during the COVID-19 pandemic has not yet been reported^25,26^.

Several possibilities exist for interpreting the remarkable decline in the prevalence of CKD, and this study observed different trends in the prevalence of CKD among different subgroups. At the time of early study design, our study predicted that outpatient clinic use would be one of the most distinct factors in the fluctuation in CKD prevalence during the early COVID-19 pandemic. In general, some people were repulsed by crowded places like hospitals or clinics at the beginning of the COVID-19 pandemic, and this study identified statistically significant difference in the prevalence of CKD on age and healthcare utilization in this society^27^. Additionally, considering that most countries, including South Korea, implemented a policy to telework during the COVID-19 pandemic (2020), many people stayed more inevitably at home than during the pre-pandemic era or were quarantined due to lockdown or strict social distancing^28^.

The changes in social life patterns since COVID-19 pandemic and the results of our study can be analyzed differently depending on the subgroup of each patient. Nevertheless, we have summarized the direction of the interpretation of this study results for several reasons. Many people may have avoided going to medical facilities for non-emergency conditions, including general outpatient visits and screening during the pandemic period^27^. This may have resulted in a decrease in the number of CKD cases detected during the pandemic, especially in vulnerable subgroups with low healthcare utilization early in the COVID-19 pandemic. However, this interpretation was not supported by the study design of our findings. This study used only two objective criteria of the CKD definition and maintained a standardized protocol that conducted health interviews and tests over 3 days in 192 primary sample units using the accompanying mobile test centers, which is itself a strength of the study. Therefore, this determined that the reduction in prevalence of CKD in our study is a substantial numerical change, and that a different direction of discussion is needed for this reduction.

Since the outbreak of the COVID-19 pandemic, it is possible that many individuals have made lifestyle changes, such as engaging in healthier eating habits, increased exercise, and stress reduction, which may have positively impacted kidney health and contributed to the decline in the prevalence of CKD^29^. Additionally, the pandemic may have influenced environmental factors, including air pollution and exposure to toxins, which could have also played a role in the decline of CKD prevalence^30^. On the other hand, these findings could be interpreted as a significant reduction in the prevalence of CKD in other groups, despite the fact that changes in lifestyle or environmental factors may not have had a significant impact on CKD prevalence among older individuals and those already receiving healthcare. However, as our study only included the early period of COVID-19 pandemic, it is premature to determine the contributing factors to the observed reduction. Therefore, it is essential to recognize that further research is needed to fully understand the exact causes for the decline in the prevalence of CKD during the early pandemic era.

Another possibility is that considerable number of deaths could have been the result of COVID-19 propagation among vulnerable group of patients who were approaching criteria for CKD died before a diagnosis of CKD. However, our result did not fit to this interpretation, because early explosive COVID-19 outbreak in Korea mainly focused on particular clusters such as churches in the city of Daegu and convalescent hospitals^31^. Due to strict social distancing and diagnostic test policies in Korea at the beginning of COVID-19 pandemic period (2020), mortality of COVID-19 was very low^31^. In that point of view, it is difficult to determine whether this significant decrease in the prevalence of CKD among young patients during the early pandemic period was directly caused by COVID-19 infection per se.

The strength of this study includes its national representative sampling design, including more than 80,000 adults, and the data examined from 2007 to 2020, including the early COVID-19 pandemic period. Through this dataset, we can interpret the nationwide longitudinal prevalence of CKD in Korea and understand the effect of the pandemic. This study had some limitations. First, there is a lack of evidence to conclude that the prevalence of CKD decreased during the COVID-19 pandemic because we included only the 1^st^ year of the COVID-19 pandemic period (transitional zone). Considering the lockdown policy due to fear of new-onset airborne infection, not only the behavior of participants but also obligatory policies could be a greater factor when interpreting patterns of medical consumption in Korea. For instance, there are more opportunities for diverse physical activities than in 2020. Therefore, if nationwide data from 2021 are gathered, additional analysis of the prevalence of CKD needs to be conducted. Second, there may have been a possibility of selection bias. However, participants were encouraged to participate in KNHANES unless there were unavoidable circumstances preventing them from doing so. The response rates of the survey were 71.1% in 2019 and 71.3% in 2020, indicating that the impact of COVID-19 on response rates was negligible. Third, the two-week timeframe for outpatient visits may be a limitation of this study, as it may be too short to capture all outpatient visits. Unfortunately, additional analyses according to the duration of follow-up were not possible because the KNHANES questionnaire only includes outpatient visits within the past two weeks as a survey item, without other visit timeframes. While regular outpatient visits of chronic patients or outpatient clinic visits for a cold or fever could lead to misclassification bias, the overall interpretation of the study is not affected. Fourth, this study used a definition of CKD based on a single eGFR value check-up, which did not meet the accurate KDIGO criteria requiring at least two eGFR values measured at least 90 days apart. Lastly, we could not analyze races and ethnicities other than those of South Koreans. Hence, further research on the impact of the pandemic in other countries is needed.

This study indicated that the national prevalence of CKD before the pandemic increased; however, the prevalence during the early pandemic period significantly decreased through a long-term trend analysis among Korean adults. Interestingly, young adults and those with low medical utilization showed significantly decreased CKD prevalence during the early pandemic. This downward slope in the prevalence of CKD may be related with lifestyle modification and environmental factors as a positive impact of COVID-19 in specific population. It is important to investigate further, as understanding the factors that influence the prevalence of CKD can help to identify healthcare policy strategies to prevent and manage the condition.

## Supplementary Information

Supplementary Information.

## Data Availability

The authors of this study declare that all main data within the paper are available. All other data are available upon reasonable request to the corresponding authors.
