# Supplementary material for: National trends in the prevalence of chronic kidney disease among Korean adults, 2007–2020
Source: Sci Rep. 2023 Apr 10;13:5831. doi: 10.1038/s41598-023-33122-1 (PMC10086041; doi:10.1038/s41598-023-33122-1)
Supplement: Supplementary file 1 — Supplementary Information. [file 41598_2023_33122_MOESM1_ESM.pdf]

National trends in the prevalence of chronic kidney disease among Korean adults, 2007-2020

Running Head: National prevalence of chronic kidney disease among Korean adults

Soo-Young Yoon,[1 ‖] Hye won Park,[2] Hyeon Jin Kim,[2 ‖] Andreas Kronbichler,[3] Ai Koyanagi,[4,5] Lee Smith,[6] Jae Il Shin,[7] Sang Youl Rhee,[2] Seung Won Lee,[8,9] Jin Sug Kim,[1], Hyeon Seok Hwang[1], Dong Keon Yon[2*], Kyunghwan Jeong[1*]

Supplementary Figure S1. Trends and prevalence of CKD among Korean adults by sociodemographic information, health related lifestyle, and medical condition, 2007-2020. Each panel shows the difference in trends and prevalence of CKD on (A) age, (B) sex, (C) residential area, (D) educational level, (E) household income, (F) BMI, (G) smoking, (H) outpatient clinic visit within two-week from the survey date, and (I) medical condition. CKD: chronic kidney disease; BMI: body mass index.

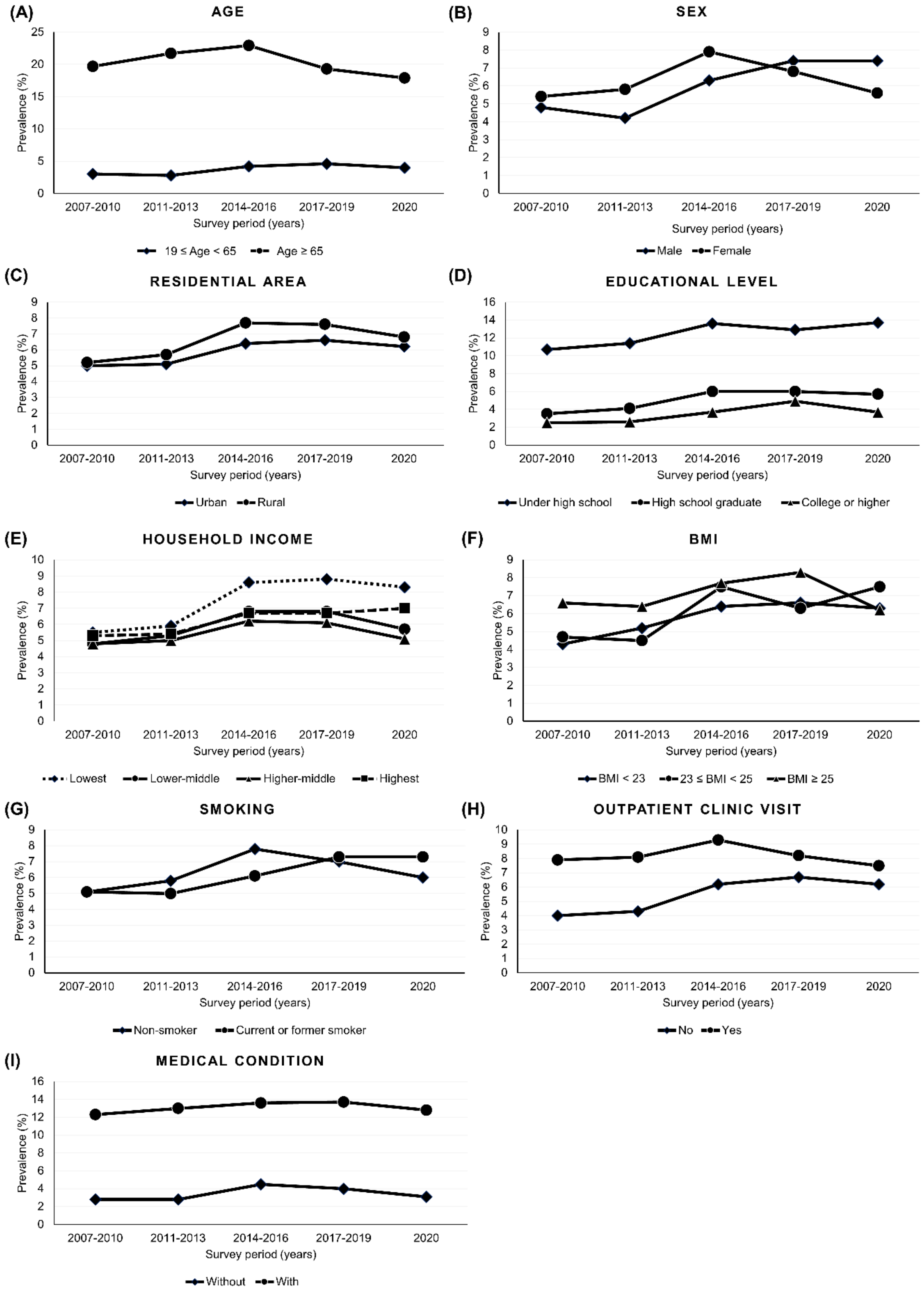
(A)
AGE
Prevalence (%)
Survey period (years)
2007-2010
2011-2013
2014-2016
2017-2019
2020
19 ≤ Age < 65
Age ≥ 65
(B)
SEX
Male
Female
(C)
RESIDENTIAL AREA
Urban
Rural
(D)
EDUCATIONAL LEVEL
Under high school
High school graduate
College or higher
(E)
HOUSEHOLD INCOME
Lowest
Lower-middle
Higher-middle
Highest
(F)
BMI
BMI < 23
23 ≤ BMI < 25
BMI ≥ 25
(G)
SMOKING
Non-smoker
Current or former smoker
(H)
OUTPATIENT CLINIC VISIT
No
Yes
(I)
MEDICAL CONDITION
Without
With
